# Editorial: Smart dietary management for precision diabetes mellitus care

**DOI:** 10.3389/fnut.2026.1757124

**Published:** 2026-01-21

**Authors:** Shaker El-Sappagh, Radwa Hassan

**Affiliations:** 1 Faculty of Computer Science, Galala University, Suez, Egypt; 2 Faculty of Medicine, Galala University, Suez, Egypt; 3 Faculty of Medicine, Cairo University, Cairo, Egypt

**Keywords:** AI in diabetes, diabetes mellitus, digital health (eHealth), mHealth, precision medicine, smart dietary management

The chronic metabolic disease known as diabetes mellitus (DM) is characterized by elevated blood glucose levels. DM can lead to serious damage to the heart, blood vessels, eyes, kidneys, and nerves over time (1). By 2040, the number of people living with diabetes is expected to increase to approximately 642 million (2). In 2016, the World Health Organization (WHO) listed diabetes as the seventh leading cause of death (3). About 7% of pregnant women have gestational diabetes, and half of those women will eventually develop Type 2 diabetes (4). Other severe chronic illnesses are also linked to diabetes. Moreover, diabetes also has a substantial socioeconomic impact (5).

Nutrition management is a critical component of the treatment of diabetes, as it plays a central role in managing blood glucose levels and overall health. However, the complexity of dietary needs, potential food–drug interactions, and the presence of comorbidities necessitate a personalized approach to dietary management (6). Recent advancements in digital health tools, particularly those leveraging artificial intelligence (AI) and machine learning (ML), offer promising solutions to create customized, data-driven dietary plans that can significantly enhance the quality of life for individuals with diabetes mellitus (7).

This Research Topic aims to explore and advance the field of smart dietary management for precision diabetes mellitus care. The Research Topic includes a wide range of objectives targeting both Type 1 and Type 2 diabetes mellitus, in addition to studies focused on gestational diabetes mellitus (GDM). They range from examinations of specific dietary patterns and intakes in individuals with diabetes, to digital applications, for diabetes dietary monitoring (image-based and mHealth), web-based dietary assessment tools, and AI-based tools (predictive analytics and generative AI) for diabetes diagnosis and management.

An observational study by Cai et al. involved 36 adults with Type 1 diabetes (T1D) using continuous glucose monitoring (CGM) devices. The study tracked participants' real-world dietary habits to analyze how the quantity of daily carbohydrate intake impacts Time in Range (TIR). The authors assessed the association between daily carbohydrate intake and glycemic control parameters in adults with T1D in free-living conditions. The authors found a strong inverse relationship between daily carbohydrate intake and glycemic control. Higher carbohydrate consumption was associated with lower TIR and higher time above range (TAR), without a significant difference in hypoglycemia [time below range (TBR)].

A case-control study (128 T2DM patients vs. 256 controls) by El-Sehrawy et al. investigated the link between diet quality scores (DQI-I and DQI-R) and the presence of Type 2 diabetes. Higher diet quality scores were significantly associated with lower odds of T2DM. Specifically, the “Adequacy” score (sufficient intake of essential nutrients) in DQI-I and the “Moderation” score (limiting sugar/fat) in DQI-R were the strongest protective factors. This reinforces that preventing diabetes requires attention not only to calorie intake but also to dietary quality and moderation.

Rouhafzay et al. conducted a scoping review of 14 calorie-counting applications and systems evaluating the computer science methodologies (segmentation, classification, volume estimation) used to automate dietary assessment. The study critically appraised existing image-based calorie-counting tools, evaluating their technical accuracy and suitability for diabetes management. The findings reveal that while deep learning has revolutionized food classification, accurate volume estimation remains a major technological bottleneck. Current AI-based nutrition applications show promise but often lack the precision required for clinical diabetes management.

Barouti et al. conducted a validation study comparing a web-based tool (“Nutrition Data”) against gold-standard interviewer-led 24-h dietary recalls (24HR) in 42 adults with T1D. The web-based program showed strong validity, with high correlations (*r* = 0.79–0.94) with the 24HRs and no significant difference in mean intakes. User acceptability was high, with 88% finding it helpful for carbohydrate counting. These findings suggest that digital, self-administered dietary tools can provide accuracy comparable to time-consuming interview-based methods and support their use in clinical practice.

Additionally, an open-label clinical trial by Murugesan et al. involving 54 women with GDM evaluated whether combining a structured food sequencing strategy with mobile health monitoring improves glycemic control and pregnancy outcomes. The intervention group showed significantly lower postprandial glucose levels, reduced LDL, and higher HDL. By simply changing the order in which food is eaten and using an app for accountability, outcomes for both mother and baby may be improved.

Among the artificial intelligence studies, Singh et al. utilized a large digital cohort (“Food and You”) to develop machine-learning models that predict postprandial glucose responses using real-world data collected via smartphones and CGM. The study found that a model using only glycemic and temporally resolved dietary data achieved high accuracy (*R* = 0.71), comparable to prior multimodal models requiring microbiome sequencing. Adding microbiome, sleep, or physical activity data did not significantly improve model performance in this real-world setting. These findings suggest that scalable personalized nutrition may be feasible without expensive or invasive clinical tests.

Another AI mini review by Deng et al. examined the evolution of AI in diabetes management, moving from traditional predictive models to emerging generative AI capacities. The review identified generative AI as a potentially transformative innovation for personalized education and synthetic dataset development. However, significant risks remain, including algorithmic bias and lack of clinician trust.

Finally, a qualitative study by Kim et al. involved experts (physicians, dietitians) evaluating weight-loss diet plans generated by ChatGPT (4.0) vs. control plans from tertiary medical centers. Only five of 67 experts correctly identified the AI-generated plan. These findings indicate that AI has reached a level of sophistication where it can generate plausible dietary plans but requires human oversight to ensure patient safety and resolve contraindications in complex medical cases.

Collectively, these studies highlight a multifaceted strategy for advancing diabetes care through smart dietary management, integrating conventional dietary therapies with innovative digital technologies. Predictive analytics enables early identification of high-risk individuals and optimization of treatment regimens, reflecting major technological progress. Despite these developments, a gap remains in integrating these technologies into routine clinical practice, underscoring the need for further research and evaluation.
